# The Dopamine Imbalance Hypothesis of Fatigue in Multiple Sclerosis and Other Neurological Disorders

**DOI:** 10.3389/fneur.2015.00052

**Published:** 2015-03-12

**Authors:** Ekaterina Dobryakova, Helen M. Genova, John DeLuca, Glenn R. Wylie

**Affiliations:** ^1^Traumatic Brain Injury Laboratory, Kessler Foundation, West Orange, NJ, USA; ^2^Department of Physical Medicine and Rehabilitation, Rutgers – New Jersey Medical School, Newark, NJ, USA; ^3^Neuropsychology and Neuroscience Laboratory, Kessler Foundation, West Orange, NJ, USA; ^4^Department of Neurology and Neurosciences, Rutgers – New Jersey Medical School, Newark, NJ, USA; ^5^War Related Illness and Injury Study Center, Department of Veterans Affairs, East Orange, NJ, USA

**Keywords:** dopamine, fatigue, mesocorticolimbic system, methylphenidate, MRI

## Abstract

Fatigue is one of the most pervasive symptoms of multiple sclerosis (MS), and has engendered hundreds of investigations on the topic. While there is a growing literature using various methods to study fatigue, a unified theory of fatigue in MS is yet to emerge. In the current review, we synthesize findings from neuroimaging, pharmacological, neuropsychological, and immunological studies of fatigue in MS, which point to a specific hypothesis of fatigue in MS: the dopamine imbalance hypothesis. The communication between the striatum and prefrontal cortex is reliant on dopamine, a modulatory neurotransmitter. Neuroimaging findings suggest that fatigue results from the disruption of communication between these regions. Supporting the dopamine imbalance hypothesis, structural and functional neuroimaging studies show abnormalities in the frontal and striatal regions that are heavily innervated by dopamine neurons. Further, dopaminergic psychostimulant medication has been shown to alleviate fatigue in individuals with traumatic brain injury, chronic fatigue syndrome, and in cancer patients, also indicating that dopamine might play an important role in fatigue perception. This paper reviews the structural and functional neuroimaging evidence as well as pharmacological studies that suggest that dopamine plays a critical role in the phenomenon of fatigue. We conclude with how specific aspects of the dopamine imbalance hypothesis can be tested in future research.

## Introduction

Fatigue is a common symptom in multiple sclerosis (MS), with up to 90% of individuals with MS reporting fatigue (1). Moreover, more than half of individuals with MS report fatigue to be their worst symptom (2). For this reason, the topic of fatigue in MS has generated a great deal of research in the domains of neuropsychology, neuroscience, and pharmacology. Other clinical populations also report fatigue including: 80% of individuals with traumatic brain injury (TBI) (3), 56% of individuals with Parkinson’s Disease (PD) (4), 99% of cancer patients (5), 88% of individuals with human immunodeficiency virus (88%) (6), as well as individuals with chronic fatigue syndrome (CFS) who experience fatigue for more than 6 months for no known psychiatric or neurological reasons (7).

Fatigue is characterized by a lack of energy, feelings of exhaustion that are unaided by sleep, and the perception that one is unable to perform mental and physical activities (8). Although fatigue has been studied extensively, in part because it affects such a wide range of clinical populations, there has been no unifying framework within which to understand fatigue. In this review, we propose such a framework, with the aim of providing structure for this developing field of study.

We propose that fatigue arises due to a dopamine imbalance within the central nervous system (CNS). One of the ultimate goals of this review is to investigate the evidence that supports the dopamine imbalance hypothesis by examining studies showing structural and functional abnormalities in areas enervated by dopamine and clinical trials showing alleviation of fatigue after dopamine medication.

The current review examines the evidence in support of the dopamine imbalance hypothesis by focusing on central fatigue, which can be experienced as both physical and mental in nature. Further, the current review builds upon a previous framework of fatigue proposed by Chaudhuri and Behan (9), which suggests that central fatigue might arise due to the “failure of the *non-motor* functions of the basal ganglia” [(9), p. 40]. This hypothesis was developed based on evidence from both animal and clinical studies, which showed the effects of basal ganglia damage to be similar to the symptoms of central fatigue. The authors emphasized subcortical pallido–thalamo-cortical interactions and urged to clarify the influence of dopamine and serotonin on fatigue, since these neurotransmitters effect the activation of the pallido–thalamo-cortical loop. In the current review, we suggest a more precise mechanism based on recent studies that fatigue might develop as a result of a dopamine imbalance.

## Dopamine: A Brief Review

**Figure 1:**
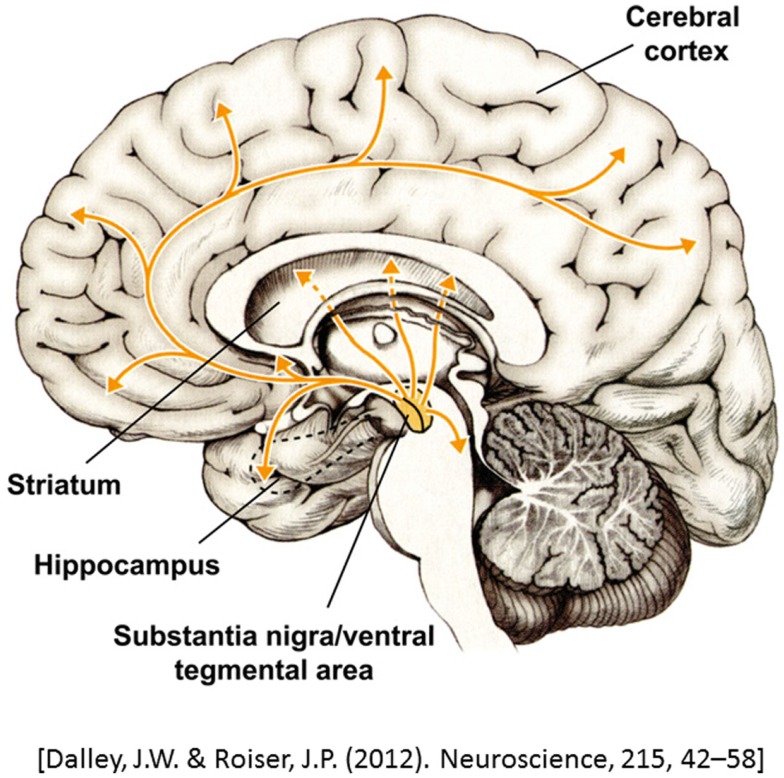
**A representation of dopaminergic projections throughout the brain**. Adapted from Dalley and Roiser (14).

Dopamine is a modulatory neurotransmitter that is termed a catecholamine due to its chemical composition. Dopamine is the most common catecholamine in the CNS (10, 11) and is a precursor to the two other catecholamines, norepinephrine and epinephrine. In the CNS, dopamine is synthesized in two subcortical brain regions, specifically, the substantia nigra pars compact (SNc) and the ventral tegmental area (VTA) (10, 12–14). Dopaminergic neurons project from the SNc and VTA to various cortical areas and thus can be segregated onto several dopaminergic pathways: (1) the nigrostriatal pathway, which links the SNc with the striatum, and (2) the mesocorticolimbic pathway, which starts at the VTA and projects to the striatum, limbic areas, and the prefrontal cortex (PFC) (10, 13, 15, 16). Finally, dopamine from a third pathway is synthesized in the hypothalamus and projects to the pituitary gland, where it is involved in the inhibition of prolactin release, a hormone that is important in immune system regulation^1^ (10) (Figure 1). Catecholamines also play an important role in the modulation of the immune system, with dopamine being synthesized and released by immune cells (17, 18).

Dopamine receptors (DRs) can be found in both the CNS and in the immune system. There are five types of DRs (D1, D2, D3, D4, and D5), subdivided into two groups: D1-like and D2-like. The D1 and D5 receptors belong to the D1-like group, while the rest of the DRs belong to the D2-like group of receptors (10, 13). These receptors have different distribution densities in the CNS, depending on the brain region. For example, more D1-like receptors are located in the PFC, while more D2-like receptors are found in the striatum. Therefore, different medications have a somewhat specific affinity for DRs and that way can have a greater effect on a specific brain region (e.g., a medication targeting D1 may have more influence on the PFC and its function than on the striatum) (19).

Dopamine has been known to play an important role in motor function. However, evidence from several past decades show that dopamine also plays a significant role in motivation and cognition. Specifically, dopamine has been shown to be involved in learning of action–outcome associations (20–22). In addition, dopamine has been shown to be involved in effortful behavior: the depletion of dopamine from the striatum or the administration of dopamine antagonists has been shown to result in the cessation of effortful reward-seeking behavior. That is, animals that learned to exert effort (e.g., climb a barrier or press a lever several times) for a larger food reward, start to prefer a smaller reward that can be obtained without effort exertion (23, 24). PFC dopamine has been shown to play an important role in working memory (25, 26). Further, increased amount of dopamine release in the striatum and the PFC has been shown to be associated with cognitive flexibility [see Ref. (27) for review].

## The Effects of Dopamine on Fatigue in the Central Nervous System

### Support from structural neuroimaging

Dopamine imbalance can be caused by changes in brain structure, particularly when structures critical for dopaminergic projections are damaged. Recent structural neuroimaging studies implicate regions of the mesocorticolimbic pathway with the fatigue experienced by several clinical populations, including those with MS. Structural impairments in the VMPFC and the striatum have been observed in fatigued individuals, suggesting a role for dopamine in fatigue (28). Pardini et al. (29) found that reduced white matter integrity in the VMPFC, a region that receives dopaminergic projections, was associated with increased fatigue in MS. In another investigation, Pardini et al. (30) assessed fatigue in individuals with TBI, finding that persons with damage localized to the VMPFC had higher levels of fatigue relative to persons with damage localized to the dorsolateral PFC or other areas of the cortex. Genova et al. (31) also showed that individuals with MS who have high fatigue have increased white matter pathology in the internal capsule, which links the striatum with the PFC (32).

Further evidence for the involvement of the mesocorticolimbic regions comes from stroke research: Tang et al. (33, 34) reported that striatal infarcts are associated with post-stroke fatigue. Additionally, magnetic resonance spectroscopy findings showed lower levels of choline concentration and *N*-acetylcholine/creatine ratio (indicative of decreased neuronal integrity) in the striatum in patients with CFS and MS, respectively (9, 35).

### Support from functional neuroimaging

Several functional neuroimaging studies also point to the involvement of mesocorticolimbic pathway in individuals with neurological damage who report fatigue. One of the earliest functional neuroimaging studies that implicated dopaminergic regions in fatigue used positron emission tomography (PET) to assess differences in brain activity at rest (i.e., without task) in individuals with MS (36). MS individuals, who scored high on the Fatigue Severity Scale (FSS) (37) exhibited reduced regional synaptic activity. That is, they exhibited lower levels of glucose metabolism in the PFC and in the striatum compared to individuals with MS who did not report fatigue (36).

Functional magnetic resonance imaging (fMRI) studies further support the dopamine imbalance hypothesis. Esposito and colleagues (38) examined the influence of fatigue in healthy individuals on resting-state network activity, i.e., task-independent activation of brain networks. Healthy individuals were scanned both at rest and while performing the *n*-back task at the beginning and at the end of: (1) a fatigue-free day and (2) a fatigue-inducing day. Participants reported increased mental fatigue and effort after performing the *n*-back task, but only at the end of the fatigue-inducing day. Moreover, reduced connectivity after the fatigue-inducing day was observed in the frontal control network that receives dopaminergic projections and is associated with executive abilities such as working memory. Other recent studies have also found altered connectivity in the mesocorticolimbic pathway in association with fatigue. Engström et al. (39) showed that MS individuals who have high fatigue show reduced mesocorticolimbic connectivity compared to healthy adults during a complex working memory task. Finke et al. (40) showed that high fatigue scores in individuals with MS were negatively correlated with resting-state mesocorticolimbic connectivity. At the same time, pharmacological studies show a reduction in fatigue following a dopamine agonist medication regimen [e.g., Ref. (41, 42)]. Connectivity between the regions of the mesocorticolimbic pathway has been shown to increase after dopamine agonist administration (bromocriptine and methylphenidate) (43–45). Taken together, these findings suggest that fatigue is associated with reduced connectivity between the regions innervated with dopamine, possibly due to reduced dopamine levels.

A potential difficulty in examining neural correlates of fatigue during task-related functional neuroimaging studies is the assessment of fatigue itself. In most of the clinical studies, fatigue is assessed with a self-report questionnaire, such as the FSS (37) or the Modified Fatigue Impact Scale (MFIS). These questionnaires provide non-specific, “global” data about the effect of “trait” fatigue on physical, social, and other activities performed during the previous weeks. However, functional neuroimaging studies are performed during well-controlled cognitive tasks that are tied to a specific time period and specific cognitive processes. A logical solution for this potential problem is to assess fatigue during task performance. Genova et al. (31) did precisely that: they asked participants with MS to rate their fatigue on a scale from 0 (not at all fatigued) to 100 (most fatigued), before and after a task-switching paradigm, a task that heavily relies on executive processing, during fMRI (46–48). These authors showed that activity in the striatum, a primary input nucleus of the mesocorticolimbic pathway, is greater in MS individuals who had higher on-task, or “state,” fatigue compared to healthy individuals (31). Similarly, a recent study reported impaired striatal functioning in individuals with CFS (49). Collectively, these functional neuroimaging findings suggest that individuals with fatigue have impaired functioning of the mesocorticolimbic pathway, likely due to a dopamine imbalance within the regions of this network.

### Support from behavioral pharmacology

A large body of evidence in support of the dopamine imbalance hypothesis is available from pharmacological studies. Several clinical trials investigated the efficacy of psychostimulant medications on fatigue in MS (50–53). Modafinil is a drug approved for treatment of narcolepsy and has been shown effective in reducing sleepiness (54). This medication might be the drug of choice for fatigue treatment in MS, since fatigue often co-occurs with (or is conflated with) sleepiness (55). However, studies that examined modafinil efficacy for fatigue have been inconclusive due to small sample sizes and methodological issues (open-label) (2, 56, 57). Amantadine has also been used to treat fatigue in MS. Amantadine is a dopamine agonists that leads to an increase in extracellular dopamine levels through promoting dopamine synthesis and blocking reuptake. A recent randomized blinded trial with four treatment groups (modafinil, amantadine, acetyl-l-carnitine, and placebo) showed amantadine to be successful in reducing fatigue (53). Of note is that Ledinek et al. (53) only included in their study MS individuals who were undergoing interferon-beta treatment (IFNβ). IFNβ is an immunomodulator that recently has been shown to aid in catecholamine synthesis (58), with dopamine being the most common catecholamine, as has been mentioned above. While this evidence is promising, the majority of clinical trials with amantadine are still underpowered and hence cannot provide conclusive evidence (51).

Several recent clinical trials have examined the efficacy of methylphenidate in treating fatigue. Methylphenidate, which has been approved for treatment of attention deficit hyperactivity disorder and narcolepsy, is a dopamine agonist that acts by inhibiting presynaptic dopamine transporters leading to suppression of dopamine reuptake (59, 60). That is, due to reuptake suppression, more dopamine remains in the synapse. Recently, a double-blind randomized placebo-controlled (DBRC) study utilizing methylphenidate showed a decrease in fatigue in 36 Parkinson’s patients (61). A DBRC trial with 60 CFS patients also showed that 20 mg of methylphenidate over 4 weeks is effective in reducing fatigue (41). Roth et al. (62) evaluated the effectiveness of a 30 mg methylphenidate dose on fatigue in 36 cancer patients in a DBRC trial, resulting in decreased fatigue after 6 weeks of treatment (62). While clinical trials with methylphenidate on MS fatigue are ongoing, the above findings support the dopamine imbalance hypothesis and suggest that restoring dopamine levels by means of dopaminergic medication results in fatigue reduction.

## Modulatory Effects of Dopamine

Unlike the two major neurotransmitters, glutamate and gamma aminobutyric acid, which have excitatory and inhibitory properties, respectively, dopamine is a neuromodulator. Studies in animals and humans show that the influence of dopamine on cognition follows an inverted “U” shape function (13, 25), with too much or too little dopamine administration leading to impaired cognitive performance. Fatigue might be subject to a similar mechanism. In the case of working memory, Gibbs and D’Esposito (63) found that healthy participants who were given a dopamine agonist (i.e., bromocriptine) showed an increase in working memory capacity (63). Harel et al. (64) showed a similar effect in individuals with MS. The authors conducted a DBRC study with 26 MS patients. Patients were classified as working memory impaired according to baseline performance on a complex task that involves working memory, processing speed, and attention. Compared to the placebo control group, follow-up task performance in the treatment group significantly improved after a single dose of methylphenidate (10 mg) taken 1 h before task follow-up (64). According to the dopamine imbalance hypothesis, administration of a dopamine agonist, such as the methylphenidate, should have lead to an increase in dopamine levels in the brain and a negative correlation between fatigue and performance; unfortunately, Harel et al. (64) did not report fatigue measures such as the FSS and the MFIS, or on-task fatigue.

Neuroimaging studies on working memory show that performance improvement in individuals with low working memory capacity is accompanied by increased connectivity between mesocorticolimbic structures increases after dopamine agonist administration (43). Vytlacil et al. (65) also showed a correlation between increased connectivity of the striatum and the midbrain nuclei (VTA and SN) and working memory performance after bromocriptine administration in individuals with low working memory capacity; an opposite pattern of results was observed in individuals with high working memory capacity. Similarly to what has been observed in the working memory literature, fatigue has been shown to be associated with reduced connectivity between mesocorticolimbic structures (39, 40). However, the effect of dopamine on mesocorticolimbic activation and connectivity in individuals with fatigue has not yet been investigated.

According to the gating hypothesis, dopamine might modulate cognition due to its interaction with other neurotransmitters in the PFC. When there is too much dopamine, the “gate” for excitatory inputs from glutamate neurons to post-synaptic PFC cells shuts down, while too little dopamine allows interference between different inputs (13, 19, 25). Similar to the gating hypothesis relating dopamine levels to cognition, the dopamine imbalance hypothesis proposes that fatigue might occur when there is too much or too little dopamine. Several studies show that while fatigue decreases with dopaminergic medication, the effect might be dose-dependent. Johansson et al. (42) observed a decrease in fatigue while participants were on a low dose of methylphenidate (5 mg), with an even greater decrease in fatigue observed when participants were on a higher dose of methylphenidate (20 mg). Similar results were obtained in hospice patients (66). Advanced cancer patients who reported high baseline fatigue, had greater fatigue reduction after 20 mg of methylphenidate (67, 68). Another DBRC trial with 109 human immunodeficiency virus participants showed methylphenidate titration to be effective in reducing fatigue. However, while some patients took the maximum dose of the medication to achieve fatigue reduction (up to 60 mg per day), other patients were able to achieve fatigue reduction with a lower dose (69).

Taken together, these studies highlight the modulatory effect of dopamine on cognition and fatigue. However, there is no evidence showing an increase in fatigue when there is too much dopamine in the CNS. Therefore, it remains to be tested if and at what dose dopamine medication ceases to be helpful in reducing fatigue. Given that dopamine is a neuromodulator that has been shown to have a variable effect on cognition (i.e., too low or too high levels of dopamine do not improve cognitive functioning), it is likely that it has a similar effect on fatigue. That is, fatigue might result from too much or too little dopamine in the brain.

## The Role of Dopamine in the Immune System

Based in large part on the evidence from the MS animal model, experimental autoimmune encephalomyelitis (EAE), MS is considered to be an autoimmune disorder of the CNS. To a large extent, the immune system depends on the functioning of the leukocytes or white blood cells. T cells are a type of white blood cell that produce an immune response, i.e., they are activated when the body needs to fight an infection. In autoimmune diseases, including MS, T cells proliferate and attack healthy cells (18), passing though the blood–brain barrier into the CNS. It has been shown that proliferating CD4^+^ cells (a type of T cells) express the D3 receptor that contributes to the destruction of dopamine neurons in the SN and generate interferon-γ, a compound that proliferates inflammation and prevents dopamine synthesis (11, 18, 70, 71). This potentially can result in decreased dopamine levels. Indeed, animal studies showed that CNS dopamine depletion by means of administration of the neurotoxin that kills dopamine cells in the SN leads to EAE exacerbation, while daily administration of a dopamine agonist, bromocriptine that has an affinity for D2 DRs (of which the striatum has a high concentration), has been shown to have beneficial effects on EAE. Treatment with bromocriptine has been shown to result in reduced severity and duration of relapses in rats with acute EAE. It also leads to the suppression of prolactin, a pituitary hormone that is inhibited by dopamine synthesized in the hypothalamus (see above) (10, 11, 17, 72). Though highly speculative, this line of reasoning suggests that fatigue might occur due to the dopamine imbalance that starts in the immune system, subsequently affecting the CNS.

IFNβ is an immunomodulatory drug approved for treatment in relapsing remitting MS. It is the first line of treatment and has been shown to prevent relapses. There are two types of IFNβ: IFNβ-1a and IFNβ-1b (73). Even though the precise mechanism of action of the IFNβ is not completely understood, it is thought that IFNβ prevents relapses and slows disability progression through retarding inflammatory processes, such as T-cell proliferation and passing of the CD4^+^ T cells through the blood–brain barrier. More importantly, recent findings also show that IFNβ treatment leads to increased production of dopamine (58, 73–75).

Given that IFNβ increases dopamine synthesis, while dopaminergic medications help increase levels of dopamine in the brain, it is possible that individuals with MS on the IFNβ treatment might benefit more from the dopaminergic fatigue treatment or even have lower levels of fatigue than individuals with MS on a different treatment. To our knowledge, there is only one study that looked at fatigue in relapsing remitting MS with IFNβ treatment (76). Melanson et al. (76) in a non-randomized open-label study showed that fatigue decreases in patients on IFNβ treatment.

## Other Fatigue Hypotheses

### Serotonin

In their seminal paper, Chaudhuri and Behan (9) called on researchers to clarify the roles of dopamine and serotonin in fatigue. Indeed, both neurotransmitters innervate the basal ganglia, with serotonergic neurons projecting to the basal ganglia from the raphe nuclei (77–79). The serotonin hypothesis developed because fatigue is a symptom of depression that is often treated with selective serotonin reuptake inhibitors. Serotonergic levels in the CNS are particularly relevant in sports medicine, as exercise has been shown to increase serotonin levels in the brain, leading to amotivation (80, 81).

A few studies in clinical populations provide evidence in support of the serotonin hypothesis, suggesting that increased levels of serotonin might lead to fatigue (82–84). For example, Pavese and colleagues used ^18^F-DOPA and ^11^C-*N, N*-dimethyl-2-(2-amino-4-cyanophenylthio) benzylamine to investigate dopamine storage capacity and serotonin transmission, respectively, in the brain of on-medication PD patients with and without fatigue (85). This PET imaging study specifically focused on the basal ganglia and limbic structures. The region-of-interest analysis revealed significant differences in serotonin transmission between PD patients with and without fatigue. However, significant differences in dopamine storage capacity between PD patients with and without fatigue were revealed only through the voxel-based analysis. Thus, the results of this study support the serotonin hypothesis and suggest that serotonin transmission might play a more important role than striatal dopamine capacity in PD-related fatigue. Unfortunately, the study of Pavese et al. had a rather small sample size (8–10 per group) rendering the results inconclusive.

Clearly, delineating the neurobiological processes underlying such a complex phenomenon as fatigue will not be simple. It is likely that the interaction of several neurotransmitters systems is involved in fatigue. Given the large body of evidence showing the mesocorticolimbic network and, in particular the basal ganglia involvement in fatigue, it is difficult to negate the involvement of serotonin neurons that also innervate the basal ganglia. However, recent studies show that decreased functioning of serotonergic receptors leads to increased functioning of dopaminergic neurons and dopamine release [for review see Ref. (86)]. Therefore, given the preponderance of evidence to date, it appears that the dopamine imbalance hypothesis of fatigue has the most support.

### Inflammatory cytokines

Recently, it has been suggested that inflammatory cytokines, compounds released by the cells of the immune system during inflammation, might be the cause of fatigue. The cytokine hypothesis developed based on the observation that fatigue co-occurs in individuals who have inflammatory biomarkers, such as tumor necrosis factor-α, interleukin-1, interleukin-6, and interferon-γ (8, 87–89).

A large body of evidence in support of the cytokine hypothesis comes from animal literature (8, 89). Indeed, animal studies show that after administration of inflammatory cytokines in the CNS, animals are less willing to exert effort in order to obtain a reward. Nunes et al. (90) showed that administration of the inflammatory cytokine interleukin-1β reduced effortful behavior in rats (decreased amount of lever presses). However, it is important to point out that effortful behavior is dopamine-dependent. Lesioning a region of the fronto-striatal network or dopamine depletion from fronto-striatal regions results in a cessation of effortful behavior (23, 91, 92). Thus, it is possible that the effect of cytokines on dopamine levels leads to fatigue, with dopamine levels being the culprit in generating fatigue and not the cytokines *per se*. Indeed, a recent study showed that methamphetamine, a psychostimulant that affects dopamine and, to a lesser extent, serotonin neurons, reduces frontocortical inflammatory cytokine levels (93), while other studies show that inflammatory cytokines have an effect on striatal functioning and dopamine release (87). Thus, such data may support the dopamine imbalance hypothesis, suggesting that the presence of inflammatory cytokines leads to dopamine imbalance.

## Summary and Conclusion

In this review, we propose that fatigue arises due to an imbalance of dopamine, a modulatory neurotransmitter, in the CNS and the immune system. Based on the evidence cited above and building upon a previous framework on fatigue (9), we propose that fatigue depends on the base levels of dopamine in the CNS. Neuroimaging studies in clinical populations with fatigue repeatedly show structural and functional impairments in regions heavily innervated by dopaminergic neurons, namely the striatum and the PFC (See Supplementary Table 1 for the list of studies). While these brain structures underlie a wide range of processes, converging evidence suggests that an imbalance in dopamine plays a key role in fatigue. Indeed, dopaminergic medication that increases dopamine levels in the brain has been shown to increase the functioning and connectivity between these regions in healthy individuals and to decrease fatigue in clinical populations (See Supplementary Table 2 for the list of studies). Thus, the dopamine imbalance hypothesis provides a unifying framework for the study of fatigue.

Given this framework, future research should be geared toward testing specific aspects of the hypothesis. While studies in cognition show that dopamine has a modulatory influence on cognitive performance, clinical trials in fatigue so far only show that dopaminergic medication decreases fatigue. Since fatigue and cognitive functions such as working memory and attention rely on a similar neural network, it is likely that dopamine has a modulatory effect on fatigue as well. Future neuroimaging and pharmacological research is needed to directly test whether this is the case. Thus, an important question is, does fatigue increase as dopamine levels increase above optimal levels? Another question that should be investigated is whether dopamine agonist administration decreases on-task or “state” fatigue in conjunction with performance improvement. This will help in linking objective measures of performance, which have been shown to be affected by dopamine, with subjective on-task fatigue ratings. Pharmacological studies should evaluate the effect of a dopaminergic medication not only in comparison to a placebo but also in comparison with non-dopaminergic medications, to show whether fatigue is differentially affected by a dopaminergic medication versus, for example, serotonergic medication.

Neuroimaging studies should focus on manipulating mesocorticolimbic network activity in controlled experimental settings. This would allow researchers to observe network functioning in fatigued individuals and to answer specific questions about the time course of network activation in a controlled environment. Investigating the time course of network activation during task performance would reveal whether it correlates with on-task fatigue. It is also worth looking at whether the increased connectivity observed after dopamine medication, which has been shown to lead to an increase in working memory performance, is associated with fatigue reduction. Answering these questions will provide valuable evidence about the underlying mechanisms of fatigue, and will ultimately allow us to develop targeted treatments for fatigue.

## Conflict of Interest Statement

The authors declare that the research was conducted in the absence of any commercial or financial relationships that could be construed as a potential conflict of interest.

## Supplementary Material

The Supplementary Material for this article can be found online at http://www.frontiersin.org/Journal/10.3389/fneur.2015.00052/abstract

## Footnotes

^1^Note that there are actually four pathways: (1) nigrostriatal, (2) mesolimbic, (3) mesocortical, and (4) hypothalamic (tuberoinfundibular). However, in human research, whether it is pharmacological or neuroimaging, it is not possible to specifically delineate the pathways, hence, here, we collapse the second and the third pathways, referring to them as the mesocorticolimbic pathway.
