# Supplementary material for: The Dopamine Imbalance Hypothesis of Fatigue in Multiple Sclerosis and Other Neurological Disorders
Source: Front Neurol. 2015 Mar 12;6:52. doi: 10.3389/fneur.2015.00052 (PMC4357260; doi:10.3389/fneur.2015.00052)
Supplement: Supplementary file 1 [file table_1.docx]

Table 1. List of neuroimaging studies cited in the review. *Abbreviations: CFS = chronic fatigue syndrome; CUD = cocaine use disorder; MS = multiple sclerosis; TBI = traumatic brain injury; PD = Parkinson’s Disease.*

| Authors | Imaging  Method | Are of Investigation | Population | N  [treatment group, control group] |
| --- | --- | --- | --- | --- |
| Engström et al., 2013 | fMRI | Fatigue | MS, Healthy Adults | 15, 10 |
| Finke et al., 2014 | fMRI | Fatigue | MS, Healthy Adults | 44, 20 |
| Genova et al., 2013 | fMRI, DTI | Fatigue | MS, Healthy Adults | 12, 11 |
| Gibbs & D’Esposito, 2005 | fMRI, pharmacology | Working Memory | Healthy Adults | 13 |
| Hesse et al., 2014 | PET | Fatigue | MS | 23, 22 |
| Konova et al., 2013 | fMRI, pharmacology | Addiction | CUD | 18 |
| Miller et al., 2014 | fMRI | Fatigue | CFS | 18, 41 |
| Mueller, et al., 2014 | fMRI, pharmacology | Functional Connectivity | Healthy Adults | 54 |
| Pardini, Bonzano, et al., 2010 | DTI | Fatigue | MS | 40, 15 |
| Pardini, Krueger et al., 2010 | CT | Fatigue | TBI | 68, 37 |
| Pavese et al., 2010 | PET | Fatigue | PD | 20 |
| Roelcke et al., 1997 | PET | Fatigue | MS | 47 |
| Tang et al., 2010 | MRI | Fatigue | Stroke | 78, 256 |
| Tang et al., 2013 | MRI | Fatigue | Stroke | 125, 375 |
| Vytlacil et al., 2014 | fMRI, pharmacology | Working Memory | Healthy Adults | 18 |
| Wallace et al., 2011 | fMRI, pharmacology | Working Memory | Healthy Adults | 13 |
| Yamamoto et al., 2004 | PET | Fatigue | CFS, Healthy Adults | 10, 10 |
