# Supplementary material for: The Dopamine Imbalance Hypothesis of Fatigue in Multiple Sclerosis and Other Neurological Disorders
Source: Front Neurol. 2015 Mar 12;6:52. doi: 10.3389/fneur.2015.00052 (PMC4357260; doi:10.3389/fneur.2015.00052)
Supplement: Supplementary file 2 [file table_2.docx]

Table 2. List of clinical trials cited in the review. *Abbreviations: acetyl-L-carnitine = ALCAR; CFS = chronic fatigue syndrome; IFNβ = interferon-β; MS = multiple sclerosis; TBI = traumatic brain injury; HIV = human immunodeficiency virus; PD = Parkinson’s Disease.*

| Authors | Type | Population | Medication | Dose [mg] | Duration [weeks] | N |
| --- | --- | --- | --- | --- | --- | --- |
| Blockmans et al., 2006 | DBRCT | CFS | Methylphenidate | 20 | 4 | 60 |
| Breitbart et al., 2001 | DBRCT | HIV | Methylphenidate, Cylert | 15, 37.5 | 6 | 144 |
| Harel et al., 2009 | DBRCT | MS | Methylphenidate | 10 | one dose | 24 |
| Johansson et al., 2014 | DBRCT | TBI | Methylphenidate | 5, 20 | 4 | 29 |
| Kerr et al., 2012 | DBRCT | Hospice patients | Methylphenidate | 10-40 | 2 | 30 |
| Ledinek et al., 2013 | Randomized, blind | MS | Amantadine, modafinil, ALCAR | 200, 200, 2g | 3 | 15 per group |
| Melanson et al., 2010 | Open-label, non-randomized | MS | IFNβ |  | 52 | 50 |
| Mendonça et al., 2007 | DBRCT | PD | Methylphenidate | 30 | 6 | 36 |
| Moraska et al., 2010 | DBRCT | Cancer | Methylphenidate | Up to 54 | 4 | 139 |
| Rammohan et al., 2002 | Single blind | MS | Modafinil | Up to 400 | 4 | 72 |
| Roth et al., 2010 | DBRCT | Cancer | Methylphenidate | Up to 30 | 6 | 32 |
| Stankoff et al., 2005 | DBRCT | MS | Modafinil | 200-400 | 5 | 115 |
